# Validity of ChatGPT in Assisting Diagnosis of Periventricular-Intraventricular Hemorrhage via Cranial Ultrasound Imaging in Very Preterm Infants

**DOI:** 10.7759/cureus.82300

**Published:** 2025-04-15

**Authors:** Huyen Quynh Trang Pham, Thi Truc Ly Vo, Thanh Thien Nguyen, Nhi T.K. Nguyen, Pham Minh Tri Nguyen, Nam-Hung Tran, Huu Dang Khoa Mai, Thu-Tinh Nguyen

**Affiliations:** 1 Department of Pediatrics, University of Medicine and Pharmacy at Ho Chi Minh City, Ho Chi Minh City, VNM; 2 Neonatal Intensive Care Unit, Children’s Hospital 2, Ho Chi Minh City, VNM; 3 Department of Neonatology, Children’s Hospital 2, Ho Chi Minh City, VNM; 4 Neonatal Intensive Care Unit, Children's Hospital 2, Ho Chi Minh City, VNM; 5 Department of Diagnostic Imaging, Children’s Hospital 2, Ho Chi Minh City, VNM; 6 Neonatal intensive Care Unit, Children's Hospital 2, Ho Chi Minh City, VNM; 7 Department of Neonatology, University Medical Center Ho Chi Minh City, Ho Chi Minh City, VNM

**Keywords:** artificial intelligence, chat gpt-4o, cranial ultrasound, periventricular-intraventricular hemorrhage, very preterm infants

## Abstract

Background

Periventricular-intraventricular hemorrhage (PV-IVH) is a common complication in very preterm infants (VPIs) and remains a significant cause of neonatal morbidity and long-term neurological impairment. Cranial ultrasound (CUS) is the standard bedside tool for early detection. This study aimed to explore the potential of ChatGPT-4o (OpenAI, San Francisco, USA), an artificial intelligence model, in interpreting cranial ultrasound images to assist in the diagnosis of PV-IVH.

Method

A cross-sectional study was conducted on 35 very preterm infants in a neonatal intensive care unit in Vietnam. The final cranial ultrasound (CUS) images, including coronal and sagittal views, were obtained within the first two weeks. Standardized coronal views through the anterior fontanelle were routinely acquired for optimal visualization, with sagittal views added as needed. The images were analyzed using the ChatGPT-4o model with a standardized diagnostic prompt and compared to interpretations by pediatric radiologists.

Results

From September 2024 to March 2025, 35 VPIs were screened for PV-IVH, of whom 16 cases (45.7%) were diagnosed with PV-IVH and 19 cases (54.3%) were not. Infants with PV-IVH required more intensive resuscitation, eight cases (50%) received positive pressure ventilation, and seven cases (43.8%) required intubation. The median postnatal age at PV-IVH detection was 10 days (interquartile range: 3.5 to 13.8 days). ChatGPT-4o correctly identified 12 out of 16 PV-IVH cases (75%) and misclassified four cases (25%) as false negatives, while accurately classifying 16 out of 19 non-PV-IVH cases (84.2%). The model achieved an area under the curve (AUC) of 0.796, with a positive likelihood ratio of 4.75 and moderate inter-rater agreement with pediatric radiologists (κ = 0.595, p <, 0.001).

Conclusions

The findings highlight the potential of accessible ChatGPT-4o in aiding early screening for PV-IVH in resource-limited settings. The model showed moderate diagnostic performance and fair-to-good agreement with specialists. However, further large-scale studies are needed.

## Introduction

Periventricular-intraventricular Hemorrhage (PV-IVH) is a serious problem commonly seen in very preterm infants (VPIs), especially those born before 32 weeks of gestation, due to the fragility of the germinal matrix vasculature [1]. PV-IVH remains a significant contributor to mortality and long-term neurological impairment in very low birth weight (VLBW) infants. A retrospective cohort study by Handley et al. (2018), which analyzed data from 44,028 preterm infants (gestational age 22+0/7 to 31+6/7 weeks) across hospitals participating in the California Perinatal Quality Care Collaborative between 2005 and 2015, reported a decline in the incidence of severe PV-IVH from 9.7% in 2005 to 5.9% in 2015, with an overall rate of 7.7% [2]. Conversely, in developing countries, the burden remains substantially higher. A multicenter study across 18 neonatal units of the Brazilian Neonatal Research Network (2013-2018) reported a PV-IVH incidence of 30.4% among 6,420 infants born between 23 and <34 weeks of gestation, with a rising trend over time [3]. Notably, severe hemorrhage accounted for 32.2% of the PV-IVH cases. In Asia, Huang et al. (2023) documented a PV-IVH incidence of 55.6% among 429 preterm neonates in China, with mild and severe hemorrhage occurring in 28.7% and 26.9% of cases, respectively [4]. In Vietnam, the incidence ranges from 16.3% to 30.9% in infants born before 37 weeks of gestation with the highest prevalence observed in those born before 32 weeks and weighing under 1500 grams [5]. Cranial ultrasonography (CUS) remains the preferred bedside imaging tool for both the diagnosis and longitudinal monitoring of PV-IVH in Neonatal Intensive Care Unit settings, thanks to its safety, portability, and non-invasive nature [6].

Currently, neonatologists are beginning to conduct point-of-care ultrasound (POCUS) [6]; however, the interpretation of the results remains dependent on the clinical experience of the physician and requires significant support from pediatric radiologists. Furthermore, there is a substantial shortage of specialized ultrasound physicians at numerous low-resource healthcare facilities. This can result in ignored diagnoses, delayed diagnoses, or inconsistencies in the diagnosis. Nowadays, artificial intelligence (AI) is increasingly applied in cranial ultrasound for preterm infants to detect brain abnormalities. According to Xie et al. (2020), a convolutional neural network (CNN)-based deep learning model was able to distinguish between normal and abnormal fetal brain ultrasound images with 96.3% accuracy, and could also identify lesion locations through heatmap visualization [7]. Xiao et al. (2023) further highlighted systems like PAICS that detect multiple brain malformations in real time with expert-level accuracy [8]. In addition, Ahmad et al. (2025) showed that deep learning models can accurately classify cranial ultrasound images to detect early brain injuries in preterm infants, highlighting AI's potential for clinical implementation in early neurological screening and diagnosis [9]. These studies show AI’s potential to support early diagnosis and reduce dependence on specialist skills, especially in low-resource settings. Despite AI’s potential, these approaches have not been widely applied or thoroughly investigated in the context of neonatal cranial ultrasound interpretation for PV-IVH. This gap is especially critical in low-resource settings with limited access to pediatric radiologists.

Integrating technology into clinical neonatology holds great promise for advancing the care of high-risk newborns. In particular, the application of artificial intelligence (AI) may contribute to standardizing imaging reports, minimizing inter-observer variability, and facilitating timely clinical decision-making during the critical early postnatal window. This is especially important in low-resource settings where access to experienced pediatric radiologists is often limited. Based on these potentials, we conducted a pilot study to evaluate the applicability of AI - specifically the ChatGPT-4o large language model - in assisting the description and early detection of PV-IVH on CUS images in preterm infants. This study aims to explore the feasibility of incorporating AI-assisted tools into routine clinical practice, particularly in healthcare settings with constrained human resources.

## Materials and methods

Ethics approval

The study received ethical approval from the Ethics Committee of Children's Hospital 2 on September 5, 2024 (Project No.: 24/24-BVND2).

Study design 

This cross-sectional descriptive study was conducted over a seven-month period, from September 2024 to March 2025.

Setting

All CUS examinations were conducted at the Neonatal Intensive Care Unit and the Department of Neonatology, Children’s Hospital 2 - a tertiary referral center for neonatal care in Southern Vietnam. The hospital has extensive experience in the management of VPIs and in performing CUS for the early detection of PV-IVH.

Participants

*Inclusion Criteria* 

All neonates with a gestational age below 32 weeks who met the following conditions were included: (i) underwent CUS within the first 72 hours after birth; (ii) had no congenital brain anomalies diagnosed prenatally; and (iii) obtained written consent from a parent or legal guardian.

Exclusion Criteria

Neonates were excluded if they had any of the following: (i) prenatally diagnosed congenital hydrocephalus, (ii) Dandy-Walker syndrome, or (iii) other major congenital brain malformations.

Variables

Primary dependent variables included the diagnostic performance metrics of the ChatGPT-4o model in identifying PV-IVH on CUS images. These metrics included sensitivity (Se), specificity (Sp), positive predictive value (PPV), negative predictive value (NPV), positive likelihood ratio (LR⁺), negative likelihood ratio (LR⁻), the area under the receiver operating characteristic curve (AUC), and Cohen’s Kappa coefficient (κ) for agreement with the gold standard.

Independent variables consisted of neonatal demographic and clinical characteristics potentially associated with PV-IVH risk, including gestational age, birth weight, sex, mode of delivery, antenatal corticosteroid administration, premature rupture of membranes, multiple gestations, and the need for resuscitation (positive pressure ventilation or endotracheal intubation).

The diagnosis and grading of PV-IVH were determined by board-certified pediatric radiologists, serving as the reference standard. Classification followed the Papile system modified by Volpe [10], with Grade I referring to hemorrhage confined to the germinal matrix or involving less than 10% of the lateral ventricle; Grade II to bleeding occupying 10-50% of the ventricle without dilation; Grade III to hemorrhage exceeding 50% with ventricular enlargement; and Grade IV to periventricular hemorrhagic infarction extending into adjacent white matter. Grades I and II were categorized as mild, while Grades III and IV were considered severe.

Study protocol

Eligible neonates were enrolled, and only the final cranial ultrasound (CUS) obtained within the first two postnatal weeks was analyzed. Coronal views were routinely acquired, with sagittal views added as needed. All CUS images were de-identified, coded, and performed by either neonatologists or pediatric sonographers. Image interpretation was independently conducted by experienced pediatric radiologists, serving as the diagnostic reference. Cases with missing or poor-quality images were excluded. Based on these interpretations, infants were categorized into two groups: PV-IVH and non-PV-IVH.

To standardize image quality, coronal scans through the anterior fontanelle were routinely obtained, allowing optimal visualization of cerebral structures including the lateral ventricles, choroid plexus, and basal ganglia [11]. When image quality was suboptimal, sagittal midline or parasagittal views were added to assess deeper structures such as the corpus callosum, ventricles, cerebellum, and Sylvian aqueduct. CUS was performed using high-frequency linear transducers (7-12 MHz), with the probe placed on the anterior fontanelle and oriented according to pediatric imaging guidelines [12].

In this study, the ChatGPT-4o model (GPT-4 Omni, developed by OpenAI, San Francisco, USA) was employed to analyze the CUS images. ChatGPT-4o is a multimodal large language model capable of processing and interpreting complex medical inputs [13]. Each de-identified ultrasound image was analyzed using a standardized diagnostic prompt constructed by the research team. The structured prompt was as follows: 

“You are a pediatric radiologist. This is a CUS image of a preterm infant (Gestational age: … weeks; Birth weight: … grams; Age at scan: … hours/days). Please evaluate the image and determine whether there are signs of periventricular-intraventricular hemorrhage. If present, grade the PV-IVH according to the Papile classification (modified by Volpe).” 

ChatGPT's responses were recorded and compared with the radiologists’ diagnoses (gold standard) to assess the model's diagnostic accuracy and agreement.

Statistical analysis

Statistical analyses were conducted using SPSS version 26.0 (IBM Corp., Armonk, USA). To improve data reliability, a double-entry process was employed, and cases with incomplete information were excluded. Categorical variables were summarized as frequencies and percentages. For continuous data, results were expressed as mean ± standard deviation (SD) if normally distributed, or as median and interquartile range (IQR) otherwise.

The diagnostic accuracy of the ChatGPT-4o model in detecting PV-IVH was assessed through sensitivity, specificity, positive predictive value (PPV), negative predictive value (NPV), positive likelihood ratio (LR⁺), negative likelihood ratio (LR⁻), and the area under the receiver operating characteristic curve (AUC), all reported with 95% confidence intervals (95% CI). The association between ChatGPT output and the radiologist-confirmed diagnosis was examined using the Chi-square test, while agreement levels were measured using Cohen’s Kappa coefficient. A p-value <0.05 was considered statistically significant.

## Results

From September 2024 to March 2025, a total of 57 preterm infants with a gestational age under 32 weeks underwent CUS screening at the Neonatal Intensive Care Unit and the Department of Neonatology, Children’s Hospital 2. Among these, 20 cases (35.1%) were diagnosed with PV-IVH. Following image quality screening, 35 infants were included in the final analysis, comprising 16 cases (45.7%) with PV-IVH and 19 cases (54.3%) without. The mean gestational age and birth weight in the PV-IVH group were 28.3 ± 1.99 weeks and 1203.1 ± 349.1 grams, respectively, compared to 29.2 ± 1.59 weeks and 1248.4 ± 281.5 grams in the non-PV-IVH group. Premature rupture of membranes was more frequently observed in the PV-IVH group, with 5/16 cases (31.3%), compared to 5/19 cases (26.3%) in the non-PV-IVH group. In contrast, the rate of complete antenatal corticosteroid administration was lower in the PV-IVH group, with 2/16 cases (12.5%) versus 7/19 cases (36.8%) in the non-PV-IVH group. A greater proportion of infants with PV-IVH required intensive resuscitation measures, including positive pressure ventilation in 8/16 cases (50%) and endotracheal intubation in 7/16 cases (43.8%). All infants received postnatal vitamin K1 prophylaxis in 35 cases (100%). The median postnatal age at first detection of PV-IVH was 10 days (IQR: 3.5 to 13.8 days). One in-hospital death was recorded in this group (see Table 1).

**Table 1:** Baseline characteristics. Baseline clinical and perinatal characteristics of infants with and without periventricular-intraventricular hemorrhage (PV-IVH). Data are presented as frequency (%), mean ± SD, or median (IQR)

Characteristics	PV-IVH (n = 16)	Non-PV-IVH (n = 19)
Male sex	7 (43.8)	11 (57.9)
Gestational age (weeks)	28.3 ± 1.99	29.2 ± 1.59
Birth weight (g)	1203.1 ± 349.1	1248.4 ± 281.5
Multiple gestations	3 (18.8)	5 (26.3)
In vitro fertilization	3 (18.8)	3 (15.8)
Premature rupture of membranes	5 (31.3)	5 (26.3)
Complete antenatal corticosteroids	2 (12.5)	7 (36.8)
Vaginal delivery	13 (81.3)	13 (68.4)
Positive pressure ventilation	8 (50)	8 (42.1)
Endotracheal intubation	7 (43.8)	8 (42.1)
Vitamin K1 administration	16 (100)	19 (100)
Age at PV-IVH diagnosis (days)	10 (3.5–13.8)	—
In-hospital mortality	1 (6.3)	0 (0)

Out of 16 cases with confirmed PV-IVH, the ChatGPT model correctly identified 12 cases (true positives: 75%) and failed to detect four cases (false negatives: 25%). Among the 19 infants without PV-IVH, ChatGPT correctly classified 16 cases (true negatives: 84.2%) and misclassified three cases as PV-IVH (false positives: 15.8%). There was a statistically significant association between ChatGPT’s predictions and the actual presence of PV-IVH (Chi-square = 12.43, df = 1, p < 0.001) (see Table 2). 

**Table 2:** Cross-tabulation of ChatGPT-4o predictions and gold standard diagnoses for PV-IVH detection. Cross-tabulation of ChatGPT-4o predictions and gold standard diagnoses in identifying periventricular-intraventricular hemorrhage (PV-IVH). Data are presented as n (%). Statistical association was evaluated using the Chi-square test. Statistically significant results are marked with an asterisk (*). A p-value < 0.05 was considered statistically significant.

ChatGPT	PV-IVH (n =16)	Non-PV-IVH (n =19)	P-value
PV-IVH detected	12 (75%)	3 (15.8%)	< 0.001*
No PV-IVH detected	4 (25%)	16 (84.2%)	

The model demonstrated a sensitivity of 75.0% (95% CI: 50.5-89.8) and a specificity of 84.2% (95% CI: 62.4-94.5). Both the positive and negative predictive values were 80.0%. The positive likelihood ratio (LR⁺) was 4.75, indicating that a positive result from ChatGPT-4o was approximately five times more likely when PV-IVH was actually present than when it was not. The negative likelihood ratio (LR⁻) was 0.297, suggesting that the model had a moderate ability to rule out PV-IVH when the result was negative (see Table 3). 

**Table 3:** Diagnostic performance metrics of ChatGPT-4o in detecting PV-IVH. The diagnostic performance of ChatGPT-4o in identifying PV-IVH was evaluated using standard metrics, including sensitivity, specificity, predictive values, and likelihood ratios. Se: sensitivity; Sp: specificity; PPV: positive predictive value; NPV: negative predictive value; LR⁺: positive likelihood ratio; LR⁻: negative likelihood ratio; PV-IVH: periventricular–intraventricular hemorrhage.

Metrics	Se	Sp	PPV	NPV	LR+	LR-
Value	75%	84.2%	80%	80%	4.75	0.297
95% CI	50.5-89.8	62.4-94.5	54.8-93.0	58.4-91.9	1.62-13.93	0.12–0.71

**Figure 1:**
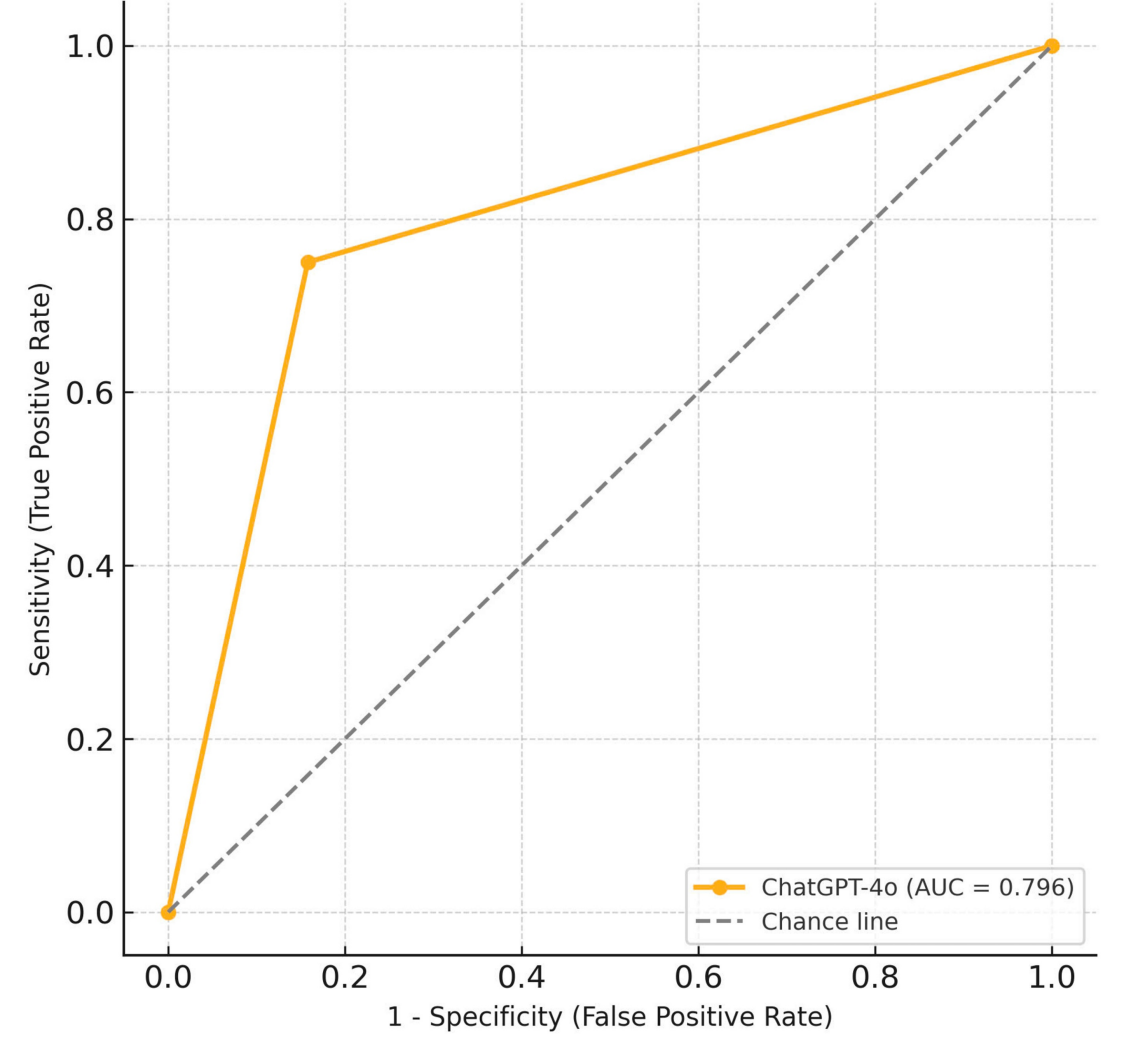
ROC curve of the ChatGPT-4o for PV-IVH diagnosis. The ROC curve illustrates the model’s diagnostic performance. The solid curve represents ChatGPT-4o (AUC = 0.796), while the dashed diagonal line represents random classification (AUC = 0.5). The model achieved a sensitivity of 75% and a specificity of 84.2%. PV-IVH: Periventricular-intraventricular hemorrhage

The area under the receiver operating characteristic curve (AUC) was 0.796, indicating fair diagnostic performance. This means that in approximately 80% of randomly selected pairs, the model could correctly distinguish between a PV-IVH case and a non-PV-IVH case (see Figure 1). 

Inter-rater agreement between ChatGPT-4o and pediatric radiologists was moderate for PV-IVH diagnosis (κ = 0.595, p < 0.001) and substantial for classification of hemorrhage severity (κ = 0.640, p < 0.001) (see Table 4).

**Table 4:** Agreement between ChatGPT-4o and expert radiologists. Inter-rater agreement between ChatGPT-4o and pediatric radiologists for detecting Periventricular-intraventricular hemorrhage (PV-IVH) and grading hemorrhage severity. κ = kappa statistic. Statistically significant results are marked with an asterisk (*). A p-value < 0.05 was considered statistically significant.

Comparison	Kappa	P-value	Level of Agreement
PV-IVH diagnosis	0.595	< 0.001*	Moderate
Severity classification	0.640	< 0.001^*^	Substantial

## Discussion

Periventricular-intraventricular hemorrhage (PV-IVH) represents a common form of brain injury among preterm neonates, particularly those born before 32 weeks of gestation. In our study, the incidence of PV-IVH was 20 cases (35.1%), with 16 cases (28.1%) classified as mild and four cases (7.0%) as severe. This underscores the high prevalence and clinical relevance of PV-IVH in this vulnerable population. Early identification of PV-IVH is essential for adjusting treatment strategies and monitoring to prevent complications and long-term sequelae. Currently, cranial point-of-care ultrasound (cPOCUS) is being increasingly utilized at the bedside by neonatologists due to its rapid accessibility and ease of use [6]. However, accurate interpretation of ultrasound images still depends heavily on individual expertise. This dependence may cause inconsistencies in diagnosis and increase the chance of missing or delaying the detection of PV-IVH. AI offers promising tools for assisting with medical image analysis, potentially improving diagnostic accuracy and reducing time to diagnosis [14]. Globally, only a limited number of studies have investigated the use of AI in CUS interpretation for neonatal brain injury, and most have focused on general abnormalities rather than specific conditions. This appears to be the first study to evaluate the application of ChatGPT-4o in detecting PV-IVH among VPIs.

The ChatGPT-4o model achieved a sensitivity of 75% and specificity of 84.2% in identifying PV-IVH. Both positive and negative predictive values (PPV and NPV) were 80%, indicating relatively high reliability for both positive and negative predictions. The positive likelihood ratio (LR⁺) of 4.75 suggests that a positive result from the model increases the probability of PV-IVH nearly fivefold. The negative likelihood ratio (LR⁻) of 0.297 indicates moderate effectiveness in ruling out hemorrhage when the result was negative. The difference between predicted and actual diagnoses was statistically significant (p < 0.001), supporting the model’s diagnostic potential.

Our study also showed that ChatGPT-4o achieved an area under the receiver operating characteristic curve (AUC) of 0.796, reflecting fair discriminatory power in differentiating between infants with and without PV-IVH. These results are encouraging, especially considering the model was not specifically trained on ultrasound data. A recent Canadian study using a deep learning convolutional neural network (CNN) to classify CUS images in extremely preterm infants reported an AUC of 0.86 and a precision-recall AUC of 0.87. Their model benefitted from training on over 4,000 images and optimization for medical imaging [9]. While our model’s performance was comparatively lower, it remains promising given the limited dataset and the use of a general-purpose language model.

The findings have important implications for clinical practice, particularly in low-resource healthcare settings or developing countries where medical personnel may face heavy workloads and limited access to pediatric radiology expertise. Although ChatGPT-4o is not specifically optimized for image analysis, it may assist in preliminary evaluations, cross-checking results, or providing early alerts when standardized ultrasound images are used. Such applications could be especially valuable in environments lacking advanced AI infrastructure or dedicated medical imaging systems, as previously emphasized by Topol in the context of high-performance, AI-augmented medicine [14].

A recent scoping review highlighted key challenges in deploying effective AI tools in low- and middle-income countries (LMICs), including limited data availability, high implementation costs, and poor adaptability to local healthcare contexts. The review authors emphasized the need for further research to evaluate the real-world effectiveness and reliability of AI in these settings [15]. The ChatGPT-4o model in our study also demonstrated good agreement with expert radiologists. The Cohen’s kappa values were 0.595 for diagnosis and 0.640 for severity classification, indicating moderate to substantial interrater reliability. These results support the feasibility of using ChatGPT-4o as an assistive diagnostic tool in clinical practice. While generalizability requires further validation in larger and more diverse populations, our findings show that even general-purpose AI models can provide clinically meaningful support when appropriately integrated into diagnostic workflows. This is particularly relevant in LMICs, where shortages of pediatric radiologists and diagnostic infrastructure are common - ChatGPT-4o may offer a practical, low-cost, and widely accessible solution to enhance neonatal care. Although final medical decisions must remain under the discretion of trained neonatologists, AI tools like ChatGPT-4o can serve as valuable complements to routine clinical decision-making in such environments.

With the growing role of AI in medicine, our study introduces a novel and practical concept: leveraging ChatGPT-4o to assist in CUS interpretation. Strengths of this study include real-world hospital data, standardized ultrasound protocols, and comparison with board-certified pediatric radiologists. However, several limitations should be acknowledged, including the small sample size, lack of model training on ultrasound images, and lower AUC compared to specialized deep learning tools. On the other hand, this simplicity is also a strength, as ChatGPT-4o is widely available and easy to implement in clinical settings with limited infrastructure. Future research should aim to validate these findings in larger cohorts, incorporate multi-timepoint data, and explore long-term neurodevelopmental outcomes to enhance the clinical utility and predictive power of AI-assisted diagnosis.

## Conclusions

This study demonstrates the preliminary feasibility of using ChatGPT-4o to support the diagnosis of PV-IVH in VPIs via CUS. The model exhibited moderate diagnostic performance and fair to good agreement with specialists. These findings underscore the potential of accessible AI tools in supporting early screening efforts, particularly in resource-limited settings. Additional large-scale research is necessary to confirm these findings and explore the extended role of AI in clinical diagnostics and long-term neurodevelopmental follow-up.

## References

[1] Novak CM, Ozen M, Burd I (2018). Perinatal brain injury: mechanisms, prevention, and outcomes. Clin Perinatol.

[2] Handley SC, Passarella M, Lee HC, Lorch SA (2018). Incidence trends and risk factor variation in severe intraventricular hemorrhage across a population based cohort. J Pediatr.

[3] Guinsburg R, de Almeida MF, de Castro JS (2016). Death or survival with major morbidity in VLBW infants born at Brazilian neonatal research network centers. J Matern Fetal Neonatal Med.

[4] Huang Z, Zhang Q, Zhu L, Xiang H, Zhao D, Yao J (2023). Determinants of low birth weight among newborns delivered in China: a prospective nested case-control study in a mother and infant cohort. J Obstet Gynaecol.

[5] Luong HT, Trac LM, Van HT (2024). Risk factors for intraventricular hemorrhage in preterm newborns at the National Hospital of Obstetrics and Gynecology. Viet J Obstet Gynecol.

[6] Singh Y, Tissot C, Fraga MV (2020). International evidence-based guidelines on Point of Care Ultrasound (POCUS) for critically ill neonates and children issued by the POCUS Working Group of the European Society of Paediatric and Neonatal Intensive Care (ESPNIC). Crit Care.

[7] Xie HN, Wang N, He M (2020). Using deep-learning algorithms to classify fetal brain ultrasound images as normal or abnormal. Ultrasound Obstet Gynecol.

[8] Xiao S, Zhang J, Zhu Y, Zhang Z, Cao H, Xie M, Zhang L (2023). Application and progress of artificial intelligence in fetal ultrasound. J Clin Med.

[9] Ahmad T, Guida A, Stewart S, Barrett N, Jiang X, Vincer M, Afifi J (2025). Can deep learning classify cerebral ultrasound images for the detection of brain injury in very preterm infants?. Eur Radiol.

[10] Mohammad K, Scott JN, Leijser LM (2021). Consensus approach for standardizing the screening and classification of preterm brain injury diagnosed with cranial ultrasound: a Canadian perspective. Front Pediatr.

[11] Cho HJ, Kim EJ, Son DW (2022). Neonatologist-performed cranial ultrasonography in the neonatal intensive care unit. Neonatal Med.

[12] Zubairi T, Khan N (2012). Central Nervous System: Brain. Atlas of Pediatric Ultrasound.

[13] (2025). Introducing GPT-4o | OpenAI. https://openai.com/index/hello-gpt-4o/.

[14] Topol EJ (2019). High-performance medicine: the convergence of human and artificial intelligence. Nat Med.

[15] Ciecierski-Holmes T, Singh R, Axt M, Brenner S, Barteit S (2022). Artificial intelligence for strengthening healthcare systems in low- and middle-income countries: a systematic scoping review. NPJ Digit Med.

